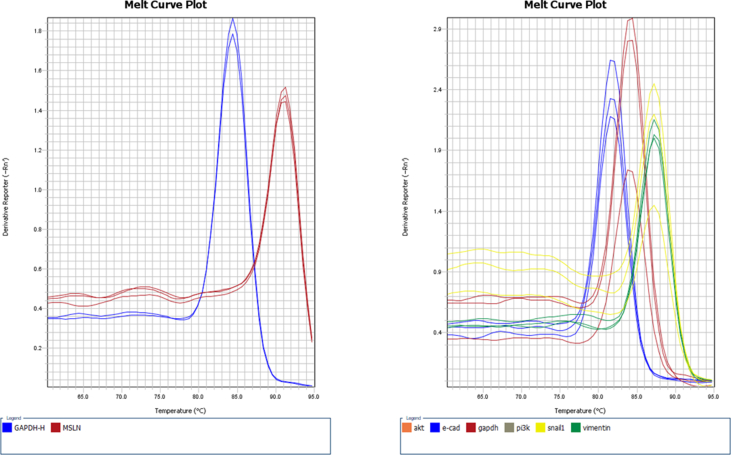# MSLN induced EMT, cancer stem cell traits and chemotherapy resistance of pancreatic cancer cells

**DOI:** 10.1016/j.heliyon.2024.e29210

**Published:** 2024-04-07

**Authors:** Jili Hu, Jia Wang, Xu Guo, Qing Fan, Xinming Li, Kai Li, Zhuoyin Wang, Shuntao Liang, Buhe Amin, Nengwei Zhang, Chaowen Chen, Bin Zhu

**Affiliations:** a Department of General Surgery, Beijing Shijitan Hospital, Capital Medical University, Beijing, 100038, China; b Department of General Surgery, Third Hospital, Peking University, Beijing, 100871, China; c Center for Biomedical Innovation, Beijing Shijitan Hospital, Capital Medical University, Beijing, 100038, China; d Department of General Surgery, Beijing Shijitan Hospital, Peking University Ninth School of Clinical Medicine, Beijing, China; e Department of Hepatobiliary Surgery, The First Affiliated Hospital of Zhengzhou University, Henan, 450052, China; f The First Affiliated Hospital of Zhengzhou University & Institute of Reproductive Health, Henan Academy of Innovations In Medical Science & NHC Key Laboratory of Birth Defects Prevention, China

**Keywords:** MSLN, Pancreatic cancer, EMT, Cancer stem cell, Chemoresistance

## Abstract

Chemoresistance is one of the main reasons for poor prognosis of pancreatic cancer. The effects of mesothelin (MSLN) on chemoresistance in pancreatic cancer are still unclear. We aim to investigate potential roles of MSLN in chemoresistance and its relationship with proliferation, epithelial-mesenchymal transition (EMT) and cancer stemness of pancreatic cancer cells. Human pancreatic cancer cell lines ASPC-1 and Mia PaCa-2 with high and low expression of MSLN, respectively, were selected. The ASPC-1 with MSLN knockout (KO) and Mia PaCa-2 of MSLN overexpression (OE) were generated. The effects of MSLN on cell phenotypes, expression of EMT-related markers, clone formation, tumor sphere formation, and pathologic role of MSLN in tumorigenesis were detected. Sensitivity of tumor cells to gemcitabine was evaluated. The results showed that adhesion, proliferation, migration and invasion were decreased significantly in ASPC-1 with MSLN KO, whereas increased significantly in Mia PaCa-2 with MSLN OE. The size and the number of clones and tumor spheres were decreased in ASPC-1 with MSLN KO, and increased in Mia PaCa-2 with MSLN OE. In xenograft model, tumor volume was decreased (tumor grew slower) in MSLN KO group compared to control group, while increased in MSLN OE group. Mia PaCa-2 with MSLN OE had a higher IC50 of gemcitabine, while ASPC-1 with MSLN KO had a lower IC50. We concluded that MSLN could induce chemoresistance by enhancing migration, invasion, EMT and cancer stem cell traits of pancreatic cancer cells. Targeting MSLN could represent a promising therapeutic strategy for reversing EMT and chemoresistance in pancreatic cancer cells.

## Graphical abstract

**Schematic diagram.** The effect of MSLN on EMT, cancer stem cell traits and chemotherapy resistance of pancreatic cancer cells: MSLN promoted the intercellular-matrix adhesion, proliferation, migration and invasion of pancreatic cancer cells; MSLN promoted pancreatic cancer cell EMT; MSLN promoted the cancer stem cell traits of pancreatic cancer cells; MSLN enhanced resistance of pancreatic cancer cells to gemcitabine.

**Figure:**
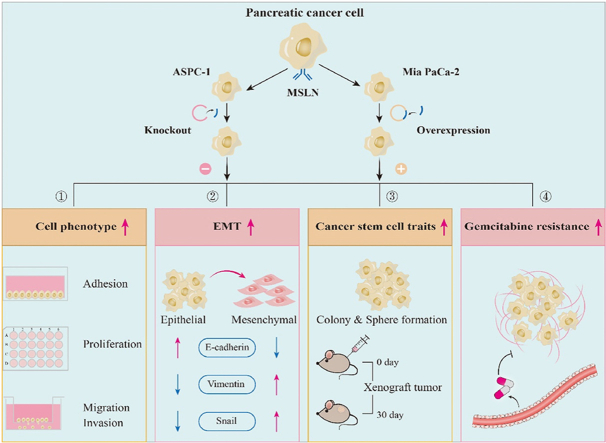

## 1 Introduction

Pancreatic ductal adenocarcinoma (PDAC), generally known commonly referred to as pancreatic cancer, is a highly lethal disease with a 5-year survival rate of less than 10%, an increasingly common cause of cancer mortality [[1], [2], [3]]. Over the past three decades, conceptual advances in diagnostic approaches, perioperative management, and systemic therapies for pancreatic cancer have been made [[3], [4], [5], [6]]. Currently, adjuvant chemotherapy following surgical resection is commonly recommended for the treatment of pancreatic cancer, with the potential to enhance the prognosis of advanced cases [1,2]. Despite this approach, the mortality rate has not experienced a substantial decrease, largely attributed to delayed diagnosis, early metastasis, and limited efficacy of chemotherapy and radiotherapy [1]. The development of chemoresistance further complicates clinical outcomes, with its multifactorial nature stemming from intricate interactions between cancer cells, cancer stem cells, and the tumor microenvironment (TME). Nevertheless, pancreatic cancer patients would benefit from targeted drugs and precision treatment.

The cell surface glycoprotein antigen Mesothelin (MSLN), which is a 40 kDa in size associated with cellular differentiation [7], is frequently highly expressed in some malignant tumors [[8], [9], [10]]. Our previous study showed that more than 90% of PDAC expressed MSLN [11]. MSLN was a predictor of PDAC mortality, superior to conventional pathologic features as a prognostic marker [[12], [13], [14]]. Additionally, MSLN has been shown to facilitate epithelial-mesenchymal transition (EMT) in lung cancer and malignant mesothelioma [15]. EMT was implicated in carcinogenesis and conferred metastatic properties upon cancer cells by enhancing mobility, invasion, and anti-apoptosis in colorectal cancer [16]. Furthermore, tumor cells originating from EMT exhibited characteristics of stem cells and demonstrated significant resistance to chemotherapy in cases of breast and colorectal cancer [17,18].

The effects of MSLN on chemoresistance and molecular mechanism underlying how MSLN promotes pancreatic cancer progression remain unclear. We hypothesize that MSLN plays a role in chemoresistance and is associated with intercellular-matrix adhesion, proliferation, invasion, metastasis and EMT of pancreatic cancer cells. Therefore, ASPC-1 with MSLN high expressing and Mia PaCa-2 with MSLN low expressing were selected. We investigated potential effects of MSLN on chemoresistance and various biological processes in pancreatic cancer cells such as proliferation, metastasis, EMT and cancer stem cell traits so as to provide a new idea for chemotherapy and targeted therapy of pancreatic cancer.

## 2 Materials and methods

### 2.1 Selection and validation of pancreatic cell lines with high and low expression of MSLN

ASPC-1 and Mia PaCa-2 cell lines derived from human pancreatic cancer were chosen based on their varying levels of MSLN expression. ASPC-1 was a gift from Peking University Health Science Center, Beijing, China. Mia PaCa-2 was provided by Laboratory of Cell Engineering, Institute of Biotechnology, Beijing, China. _2_ASPC-1 cells were cultured in RPMI 1640 growth medium from Hyclone, USA, supplemented with 10% FBS from Gibco, USA, and 100 U/ml of penicillin and 100 pg/ml of streptomycin from Gibco, USA. Mia PaCa-2 cells were cultured in DMEM growth medium from Hyclone, USA, supplemented with 10% FBS, 100 U/ml of penicillin, and 100 pg/ml of streptomycin. All cell lines were maintained in a humidified incubator at 37 °C with 5% CO2.

The expression of MSLN in pancreatic cancer cells was confirmed by qPCR and Western blot. See below for details.

### 2.2 Generation of MSLN knockout (KO) cell line by gene editing

The MSLN gene sequence was retrieved from Genbank and Ensemble databases. gRNAs for CRISPR-Cas9 were designed using the http://crispr.mit.edu/ website, synthesized, and cloned into the LentiCRISPR-v2 plasmid (courtesy of Shuntao Liang). The gRNA sequence is detailed in Table 1. ASPC-1 cells were transduced with Lentivirus. This CRISPR/Cas9 plasmid co-expresses Cas9 and GFP, allowing for the initial sorting of GFP-expressing cells via flow cytometry (MoFlo XDP, Beckman Coulter, USA), followed by the establishment of single-cell clones. After expansion, the resultant cells were subjected to analysis through Sanger sequencing and Western blot. MSLN levels in MSLN-knockout (AKO), empty vector–transfected (ANC) and wild type ASPC-1 (ACON) cell lines were compared.

**Table 1:** The sequences of three sgRNA primers.

sgRNA name	Sequences of gRNA
sgRNA 1-71	GTCCCACAGGACCCCAACAG
sgRNA 2-61	ACAGACCATGGCCTTGCCAA
sgRNA 3-68	ACCCACCTAACATTTCCAGG

### 2.3 Generation of MSLN overexpression (OE) cell line by lentiviral transfection

Pancreatic cancer cell lines were transfected with lentiviral particles, and stable MSLN-expressing cell lines were obtained by selecting with 3 μg/ml puromycin (Sigma,USA). Cloning plasmid of MSLN cDNA was supported by Sino Biological Inc. Beijing, China. Lenti E6-MSLN plasmid vector was constructed with a Bam HI enzyme cleavage site before the start codon and an XBaI enzyme cleavage site after the termination codon. The sequences of Lenti E6-MSLN plasmid vector primers were shown in Table 2. Lenti E6-MSLN plasmid vector were nucleofected into Mia PaCa-2 cells by Lentivirus. The top 5% of GFP-positive cells were isolated using flow cytometry (MoFlo XDP, Beckman Coulter, USA). The expanded cells exhibiting MSLN overexpression were then analyzed by Western blotting, with GAPDH serving as an internal reference. MSLN levels in MSLN overexpression (MOE)), empty vector–transfected (MNC) and wild type Mia PaCa-2 (MCON) cell lines were compared.

**Table 2:** The sequences of Lenti E6-MSLN plasmid vector primers.

Primer name	Sequences of primer (5′ to 3′)
XBaI + M	TTGCCGCCAGAACACAGGACCGGTTATGGCCTTGCCAACGGCTCG
BamHI-P2A-M-	AGAGAGAAGTTTGTTGCGCCGGATCCGGCCAGGGTGGAGGCTAGG

Primers were synthesized by Tsingke Biotechnology Co., Ltd. Beijing.

### 2.4 Evaluation of intercellular-matrix adhesion and proliferation ability of pancreatic cancer cells

**Adhesion assay:** Cancer cells were placed in 24-well plates a concentration of 1 × 10^4^ cells per well on Matrigel-coated surfaces (Corning, USA). Following incubation at 37 °C for 2, 4, 8, and 12 h, cells that did not adhere were removed by washing three times with PBS. After that, the cells that stuck were treated with 4% paraformaldehyde for half an hour and then colored with 0.1% crystal violet for 10 min at ambient temperature. Subsequently, the crystal violet solution was washed off thrice with ddH_2_O. Adherent cells were quantified by counting the average number of cells in 5 randomly selected high-magnification ( × 100) fields using a microscope (ECLIPSE TS100, Nikon, Japan). Each experiment was replicated in triplicate wells across three independent trials.

**Cell proliferation assay:** Cell proliferation was assessed by performing a cell proliferation assay with the cell counting kit-8 (Dojindo, Tokyo, Japan), according to the manufacturer's instructions. Cultured cells were placed in 96-well dishes with 1 × 10^3^ cells in each well and left to incubate for 1–5 days. Following the removal of the culture medium, a 10% CCK8 solution was introduced into each well, and the cells were then placed in an incubator at 37 °C for a duration of 2 h. The light absorption was subsequently assessed at a 450 nm wavelength with the Tecan Spark 10 M luminometer (Tecan Group Ltd., Switzerland). Each sample was assessed in six replicate wells.

### 2.5 Evaluation of migrating and invasion ability of cells

**Wound healing assay:** 1 × 10^6^ cancer cells were placed in a 6-well plate and cultured in an CO_2_ incubator. A wound was created by scratching cancer cells with a 10 μl pipette tip when reached confluency. PBS was used to wash away cell debris. Serum-free medium was added for culture. The cell-free area was photographed at 0, 24, 48, 72, and 96 h using a microscope equipped with a digital camera (ECLIPSE TS100, Nikon, Japan). Image J software from the National Institutes of Health, USA, was employed to measure the wound closure area. The experiments were conducted in triplicate dishes and repeated three times.

**Transwell migration assay:** Transwell filters were utilized for cell migration analysis. Specifically, uncoated polycarbonate filters with an 8.0-μm pore size (Corning Costar, USA) were employed. To begin the experiment, 2 × 10^4^ cells were seeded into the upper chamber in 1% FBS media, while 10% FBS media was added to the lower chamber. Following a 24 h incubation period at 37 °C, the upper membrane surface was gently wiped with a cotton swap. Subsequently, the cells located underneath were treated with 4% paraformaldehyde for half an hour and then colored with 0.1% crystal violet for 10 min. Cell migration was measured by tallying the mean quantity of cells that had migrated in 5 randomly selected fields under a high magnification ( × 400) microscope. Each experiment was triplicated and conducted independently three times.

**Transwell invasion assay:** Cancer cells (5 × 10^4^ cells/well) in 1% FBS media were seeded onto the upper chambers of 8.0-μm Transwell Permeable Supports (Corning, USA) pre-coated with Matrigel (Corning, USA). 10% FBS media was added in the lower chambers. Cells were incubated at 37 °C for 24, 48, and 72 h, followed by fixing the cells on the lower surface with 4% paraformaldehyde for 30 min and staining with 0.1% crystal violet for 10 min. Cell invasion was evaluated by counting the mean number of cells that had migrated in 5 randomly chosen high-magnification ( × 400) areas. Each assay was tested in triplicate wells of three independent experiments.

### 2.6 PCR assay of MSLN and EMT-related markers

Total RNA was extracted from cell lines using Trizol (Sigma, USA). RNA isolation followed the manufacturer's protocol using TRIzol reagent. Subsequently, 1 μg of RNA was reverse transcribed into cDNA using the ReverTra Ace qPCR RT Kit (Toyobo, Osaka, Japan). Real-time qPCR was carried out on an ABI Prism® 7500 Fast Real-Time PCR system (Applied Biosystems, USA). Gene expression levels were determined using the 2-ΔΔCt method, with Glyceraldehyde-3-phosphate dehydrogenase (GAPDH) used as the housekeeping gene. The sequences of primers were shown in Table .3 and qPCR conditions in Table .4 qPCR reactions were repeated three times. Independent experiments were performed in triplicate.

**Table 3:** Sequences of primers.

Primer name	Sequences of primer
MSLN-F	CCCGTTTCTTCTCCCGCATCAC
MSLN-R	TCCCAGAGCCCGCACATCAG
E-Cadherin-F	GCCATCGCTTACACCATCCTCAG
E-Cadherin-R	CTCTCTCGGTCCAGCCCAGTG
Vimentin-F	TGAATGACCGCTTCGCCAACTAC
Vimentin-R	CTCCCGCATCTCCTCCTCGTAG
Snail1-F	CCTCGCTGCCAATGCTCATCTG
Snail1-R	GCTCTGCCACCCTGGGACTC
GAPDH-F	TGACATCAAGAAGGTGGTGAAGCAG
GAPDH-R	GTGTCGCTGTTGAAGTCAGAGGAG

Sangon Biotech Co., Ltd. Shanghai synthesized the primers.

**Table 4:** qPCR conditions.

Procedure	Cycles	Temperature (°C)	Time (s)
Predenaturation	1	95	30
PCR	40	95	5
		60	30
		95	15
Dissolve	1	60	30
		95	15

### 2.7 Western blot assay of MSLN and EMT-related markers

Whole-cell extracts were prepared by RIPA lysis buffer (Solarbio, China) with phenylmethanesulfonyl fluoride (PMSF) and protease inhibitor cocktail (Solarbio, China). After separating cells by SDS-PAGE, lysates were transferred onto PVDF membranes (Bio-Rad, USA) followed by blocking with 5% non-fat milk in the Western blotting procedure. Following this, the membranes were subjected to blocking with 5% non-fat milk at room temperature for a duration of 2 h. Subsequently, the membranes were incubated with specific primary antibodies targeting human MSLN, Vimentin, Snail, E-cadherin, and GAPDH (obtained from Cell Signaling Technology, USA) at 4 °C overnight. Finally, the membranes were examined using Dylight 680 or Dylight 800 linked secondary antibodies (Cell Signaling Technology, USA). An internal reference was utilized in the form of GAPDH. The bands were observed using an Odyssey® CLX two-color infrared laser imaging system (Li-cor, USA).

### 2.8 Evaluation of cancer stem cell traits of pancreatic cancer cells

**Plate cloning assay:** Cell cloning experiment involved seeding cells in 6-well dishes at a concentration of 500 cells per well, with three replicate wells per group. After a two-week incubation period, the cells were washed three times with PBS and subsequently dyed with 0.1% crystal violet for a duration of 10 min. The number of cell colonies (≥50) was counted under a microscope. Colony formation efficiency was calculated as the ratio of the number of colonies to the number of inoculated cells, multiplied by 100%.

**Soft agar clone formation assay:** In the soft agar cloning assay, cancer cells were mixed with 7 g/L agar to reach a final concentration of 3.5 g/L, with 1 × 10^6^ cells per well in 1 ml of culture medium. The cell-agar mixtures were then plated into 6-well plates containing 12 g/L agar in culture medium without delay. Following a two-week incubation period, colonies were viewed under a light microscope. The colony formation rate was determined by dividing the number of colonies formed by the number of cells initially inoculated, then multiplying by 100%.

**Tumor sphere formation assay:** The experiment for tumor sphere formation was conducted in a non-adherent and serum-free environment. In short, 1 million cells were placed in DMEM/F12 (Gibco) serum-free medium with 10 ng/ml epidermal growth factor (Peprotech, USA), 10 ng/ml basic fibroblast growth factor (Peprotech, USA), and 1 × B27 (Gibco, USA) in ultra-low adherent 6-well plates (Corning, USA). After a 14-day incubation period, tumor spheres were observed using a light microscope. The spheres were collected by gentle centrifugation to assess their ability to self-renew, then separated into individual cells, filtered, and finally cultured in the same conditions to produce secondary spheres.

**Establishment of xenotransplantation model of pancreatic cancer:** Female immunodeficient NuNu mice (6–8 weeks old), obtained from Vital River Laboratory (Beijing, China), were kept in a sterile environment at the Animal Laboratory of Beijing Shijitan Hospital, Capital Medical University. The building kept a consistent temperature (23 ± 1 °C), humidity (55 ± 5%), and followed a 12-h light/dark schedule. The mice had ad libitum access to water and food. They were randomly assigned to four groups (n = 5/group) and received injections of the corresponding cells: AKO, ACON, MCON, and MOE. The single cell suspension (100 μl) with 1 × 10^6^ cells in 50% Matrigel (Corning, USA) was subcutaneously transplanted into the right back of a mouse. Each day, the mice were observed for symptoms of illness, including decreased weight, hunching, lack of grooming, and red eye discharge. Tumor diameter was measured daily using digital electric calipers, and tumor volume was calculated twice a week after inoculation using formula (V (mm^3^) = width^2^ × length/2). After 30 days, the mice were anesthetized, sacrificed, and tumors were completely excised. Mice were euthanized if the tumor size reached a maximum diameter of 20 mm, if they lost 20% or more of their body weight, or if they displayed signs of compromised health or distress.

### 2.9 The half inhibitory concentration (IC_50_) of gemcitabine

AKO, ANC, ACON, MOE, MNC, MCON cell lines were used to clarify MSLN could promote resistance to gemcitabine in pancreatic cancer cells or not.

CCK-8 assays (Dojindo, Tokyo, Japan) were used to determine gemcitabine's IC50. Cancer cells (5 × 10^3^ cells/well) were seeded in 96-well plates and left to incubate at 37 °C overnight. Various doses of Gemcitabine hydrochloride (Nanjing Zhengda Tianqing Pharmaceutical Co., LTD, China) were used to treat cancer cells for 48 h at 37 °C. Then, 10 μl of CCK-8 solution was added to each well and incubated at 37 °C for 2.5 h. Subsequently, the absorbance was determined using a Tecan Spark 10 M luminometer (Tecan; Tecan Group Ltd., Mannedorf, Switzerland) at 450 nm. Cytotoxicity was determined as a percentage of viability. Cell viability (%) = absorbance of sample cells/absorbance of untreated cells × 100. The IC_50_ was calculated by using a nonlinear fit with GraphPad Prism 8.0 software, which was used as an indicator to evaluate the sensitivity to gemcitabine. Each sample had six duplicate wells, and the experiments were conducted three times.

### 2.10 Statistical analysis

Prism 8 (GraphPad Software, San Diego, USA) and SPSS Statistics 26.0 (IBM software) were utilized for data analysis. The findings were presented as the average plus or minus the standard deviation. The Student's *t*-test was utilized to assess variances between two groups, whereas comparisons involving three or more groups utilized one-way repeated measures ANOVA, one-way ANOVA with Tukey's post hoc test, or Kruskal-Wallis test with Dunn's post hoc test for nonparametric data. A p value less than 0.05 was deemed to be statistically significant. Asterisks denoted significance levels as follows: *, p < 0.01; **, p < 0.05; ***, p < 0.001; ****, p < 0.0001, unless stated otherwise.

## 3 Results

### 3.1 MSLN knockout and overexpression cell lines

ASPC-1 highly expressed MSLN, while Mia PaCa-2 low expressed MSLN as measured by qPCR and Western blot (Fig. 1a and b).

**Fig. 1:**
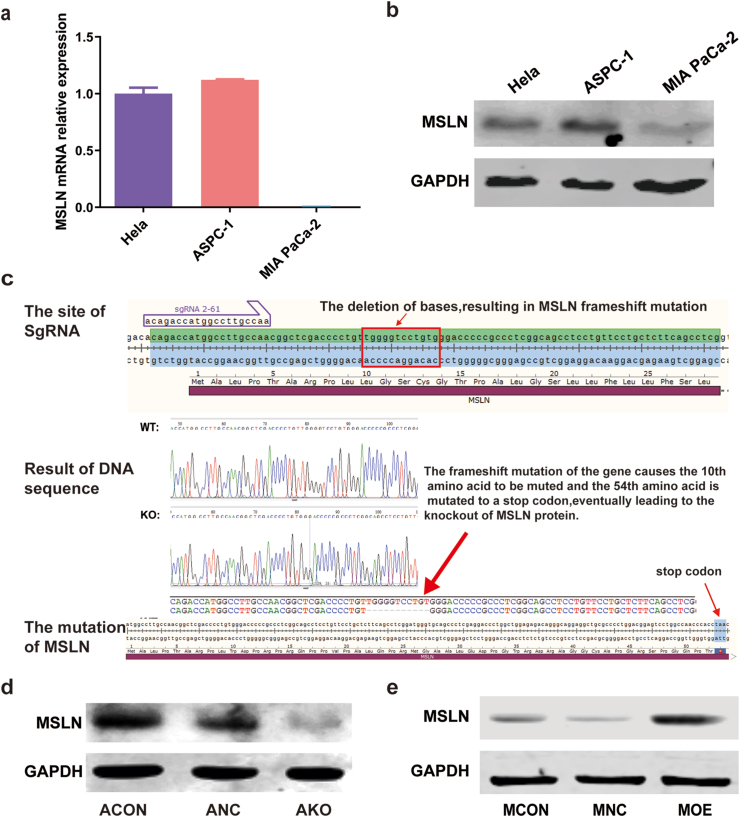
MSLN knockout and overexpression cell lines were generated. The high expression of MSLN in ASPC-1 and the low expression in Mia-PaCa-2 cell lines were verified by qPCR(a) and Western blot(b, Fig. S1). Mean ± SD was used to present all data. DNA sequencing revealed a frameshift mutation in the gene resulting in the knockout of MSLN protein. The expression of MSLN protein was confirmed in ASPC cells through Western blot analysis(d, Fig. S2). Verification of MSLN protein expression in Mia PaCa-2 cells by Western blot (e, Fig. S3). ACON: wild type ASPC-1 cell line; ANC: empty vector–transfected cell line; AKO: MSLN knockout cell line. MCON: wild type Mia PaCa-2 cell line; MNC: empty vector–transfected cell line; MOE: overexpression cell line.

A monoclonal cell line (vector transfected with sgRNA1-71+sgRNA 2–61) was successfully knocked out by 11 bases TG GGG TCC TGT, resulting in a frameshift mutation of MSLN gene. As shown in Fig. 1c, the frameshift mutation caused loss of the 10th amino acid and shift of the 54th amino acid into a stop codon, and eventually led to KO of MSLN. The loss of MSLN expression in AKO was confirmed by DNA sequencing and Western blot. The expression of MSLN in ANC was similar to that in ACON (Fig. 1d). The MSLN cDNA clone plasmid was successfully transfected into Mia PaCa-2 cells (MOE) by lentiviral transfection. MSLN was highly expressed in MOE compared with MCON and MNC (Fig. 1e).

### 3.2 MSLN promoted cell intercellular-matrix adhesion, proliferation, migration and invasion

MSLN enhanced intercellular-matrix adhesion (Fig. 2a–d). MSLN strengthened cell proliferation (Fig. 2e and f). MSLN was found to enhance cell migration based on the results of the wound healing assay (Fig. 3a–d) and transwell migration assay (Fig. 3e–h). Based on transwell invasion assay, MSLN promoted invasiveness (Fig. 4a–d). Vector–transfected cell lines were similar to those of wild type cell lines. MSLN promoted biological behaviors of pancreatic cancer cells, such as intercellular-matrix adhesion, proliferation, migration and invasion.

**Fig. 2:**
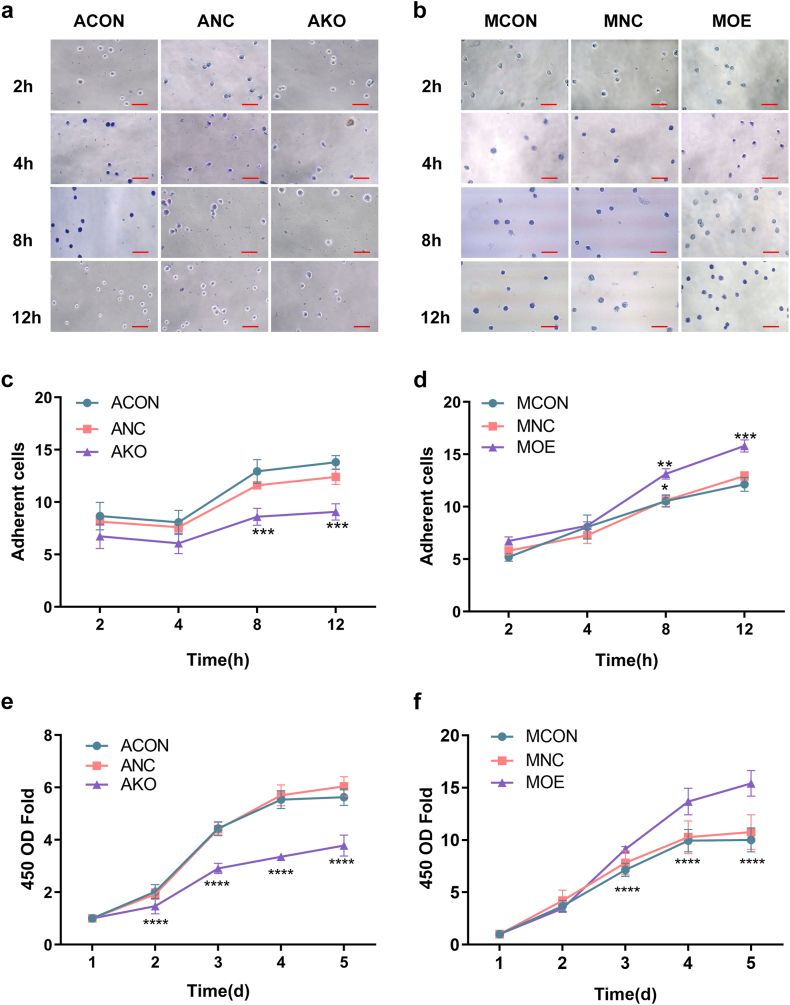
MSLN promoted the intercellular-matrix adhesion and proliferation of pancreatic cancer cells. Cell adhesion at different time points (a–d), the quantity of cells adhering was determined by counting from 5 randomly chosen fields using a microscope. Cell proliferation assessed using the CCK-8 assay (e, f), with results shown as mean ± SD. Scale bar = 100um. *P* < 0.01 *p* < 0.05, *p* < 0.001, and *p* < 0.0001 were denoted by single (*), double (**), triple (***) and quadruple (****) asterisks respectively.

**Fig. 3:**
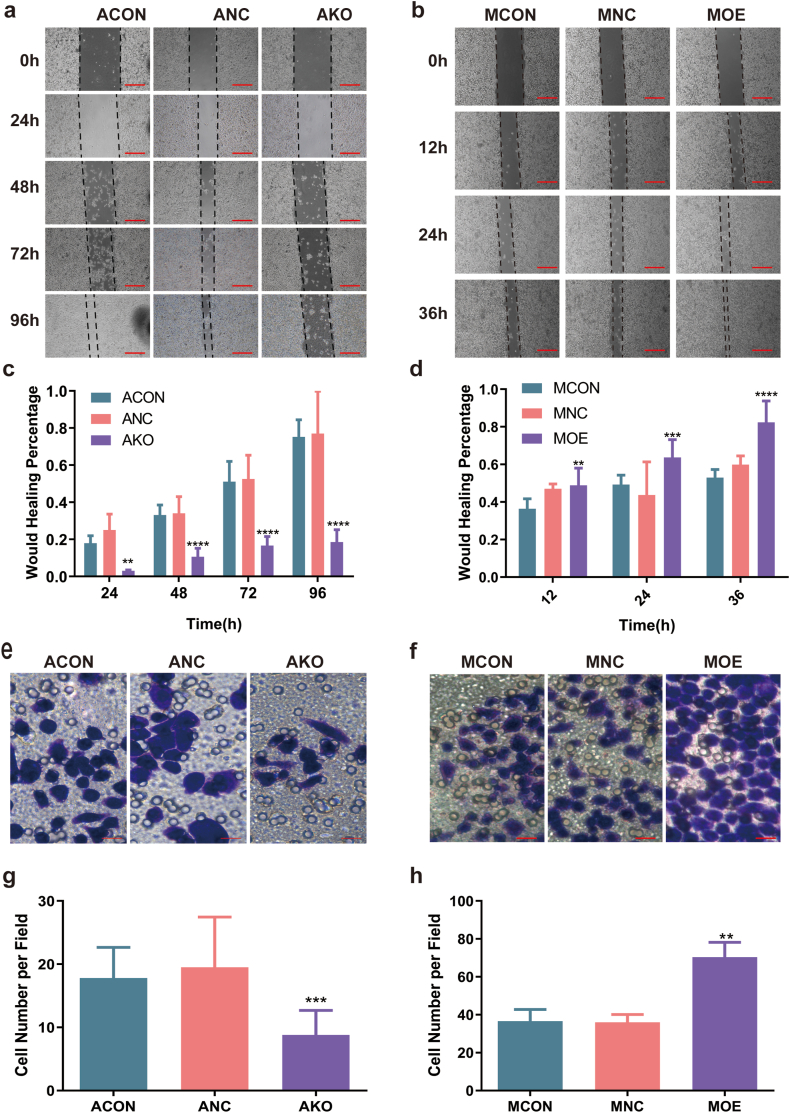
MSLN promoted the migration of pancreatic cancer cells. Migration of ACON, ANC, AKO and MCON, MNC, MOE assessed using a wound healing assay at specified time points post-scratching (a–d), and transwell migration assay (e–h). The distance of the wound healing area was measured by Image J software, each scratch is measured 3 times. The number of migrated cells was determined by counting cells in five randomly selected fields under a microscope. Scale bar _(a,b)_ = 400um, Scale bar_(e,f)_ = 100um. All data were presented as mean ± SD. *p* < 0.01 *p* < 0.05, *p* < 0.001, and *p* < 0.0001 were denoted by single (*), double (**), triple (***) and quadruple (****) asterisks respectively.

**Fig. 4:**
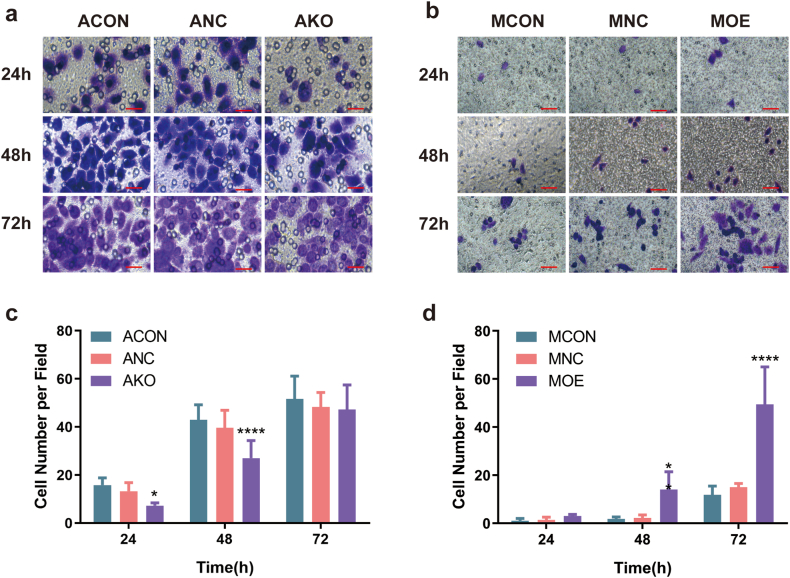
MSLN promoted the invasion of pancreatic cancer cells. Invasion of ACON, ANC, AKO and MCON, MNC, MOE, detected by transwell invasion assay (a–d). Five fields were randomly selected under the microscope to count the number of invased cells. Scale bars = 100μm. Data were presented as mean ± SD. *p < 0.05, **p < 0.01,***p < 0.001,****p < 0.0001.

### 3.3 MSLN promoted the EMT of pancreatic cancer cells

The mRNA expression levels of EMT-related markers were measured by qPCR assay. E-Cadherin mRNA expression was decreased in cells with high MSLN expression, while increased in cells with low MSLN expression (Fig. 5a and b). By contrast, the levels of Vimentin and Snail mRNA expression was elevated in cells exhibiting high MSLN expression and reduced in cells with low MSLN expression, as shown in Fig. 5c–f. EMT-related markers were validated by Western blot. E-Cadherin protein was decreased in cells with high MSLN expression, while increased in cells with low MSLN expression (Fig. 5g). By contrast, Vimentin and Snail protein was increased in cells with high MSLN expression and decreased in cells with low MSLN expression (Fig. 5g). Therefore, MSLN could potentially trigger EMT in pancreatic cancer cells.

**Fig. 5:**
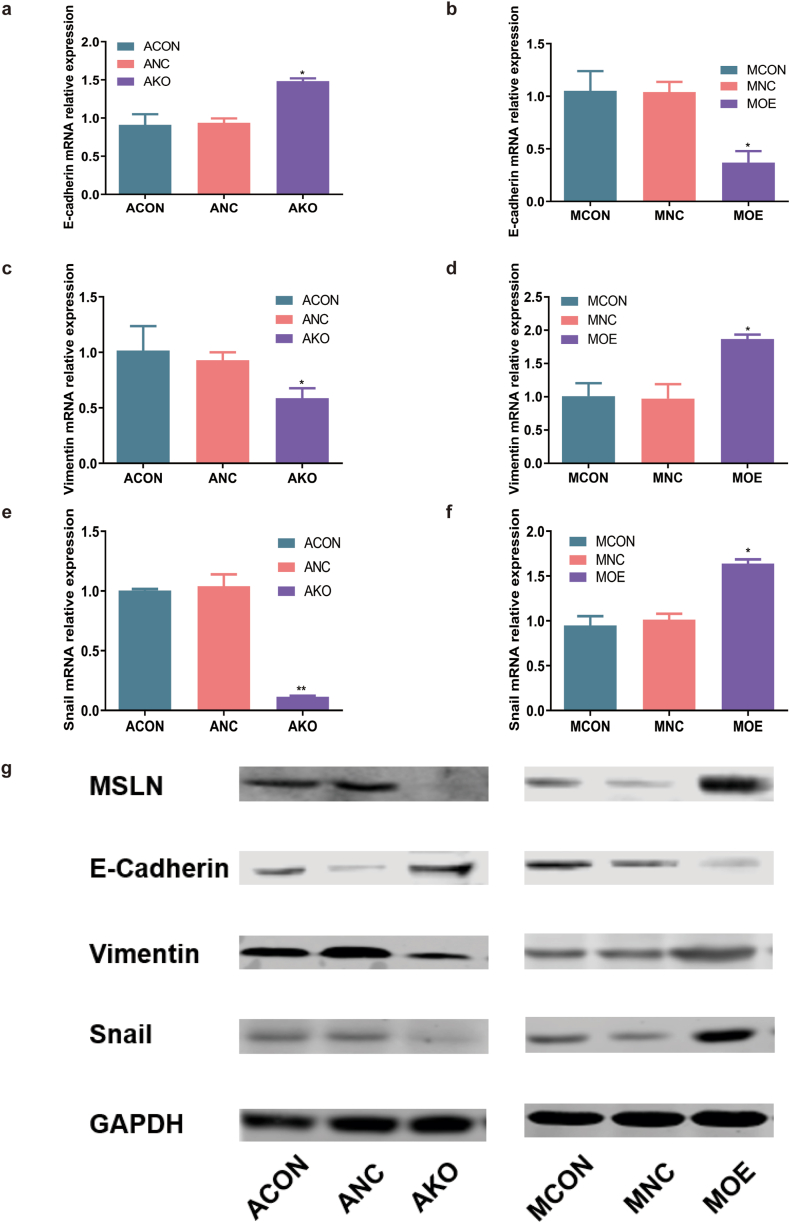
MSLN promoted the EMT of pancreatic cancer cells: Knockout of MSLN(Fig. S4) up-regulated E-Cadherin whereas down-regulated Vimentin and Snail; Overexpression of MSLN(Fig. S3) up-regulated Vimentin and Snail whereas down-regulated E-Cadherin. Evaluation of EMT by mRNA (a–f) and protein (g) level of E-Cadherin (Figs. S5 and S6), Vimentin(Figs. S7 and S8) and Snail(Figs. S9 and S10). GAPDH served as a reference gene or housekeeping gene. Data are presented as mean ± SD, n = 3, *P < 0.05, **P < 0.01.

### 3.4 MSLN promoted cancer stem cell traits

Clone formation and tumor sphere formation were decreased in MSLN low-expressing cells whereas increased in MSLN high-expressing cells (Table 5; Fig. 6a–f). *In vivo*, mice grew visible tumors 10 days after inoculation. Tumor volume was gradually increased with time. The tumors generated by MSLN low-expressing cells grew slower than those by MSLN high-expression cells. The xenograft tumor weight and volume were lower and smaller in nude mice generated by MSLN low-expressing cells than those by MSLN high-expressing cells (*P* < 0.05) 30 days after inoculation. MSLN could promote cancer stem cell characteristics of pancreatic cancer (Table .6, Fig. 6g–j**).**

**Table 5:** The rate of clone formation.

Groups	Clone forming rate (%)	*P* value
ACON	3.40%	*P* < 0.05
ANC	3.80%	
AKO	1.80%	
MCON	2.20%	*P* < 0.05
MNC	2.40%	
MOE	4.20%

**Fig. 6:**
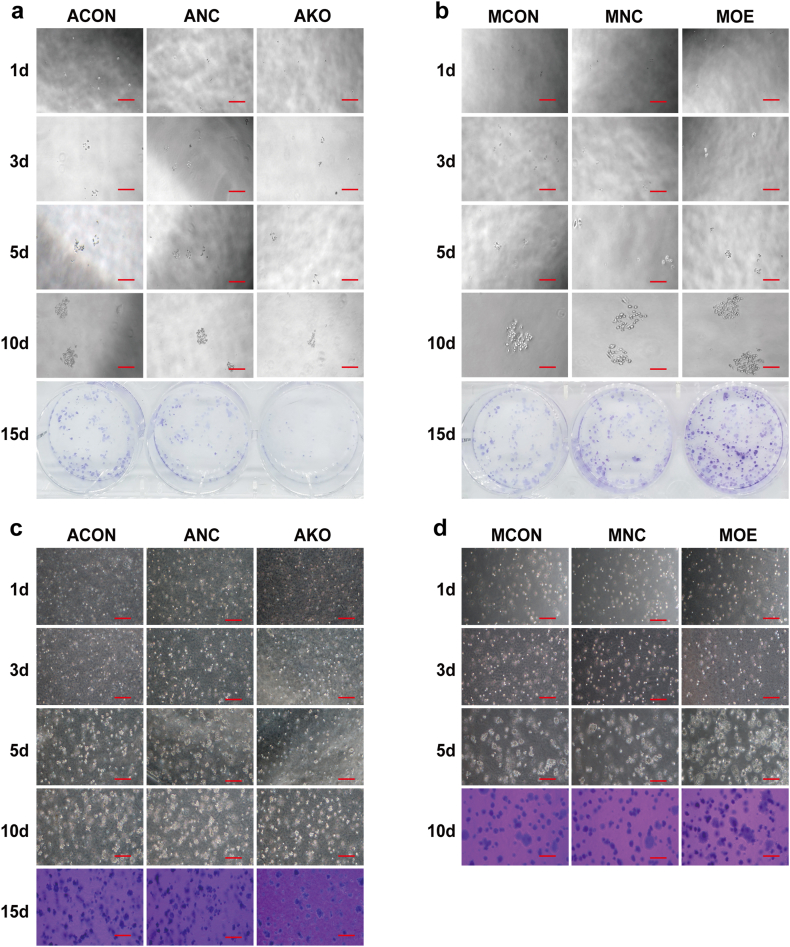

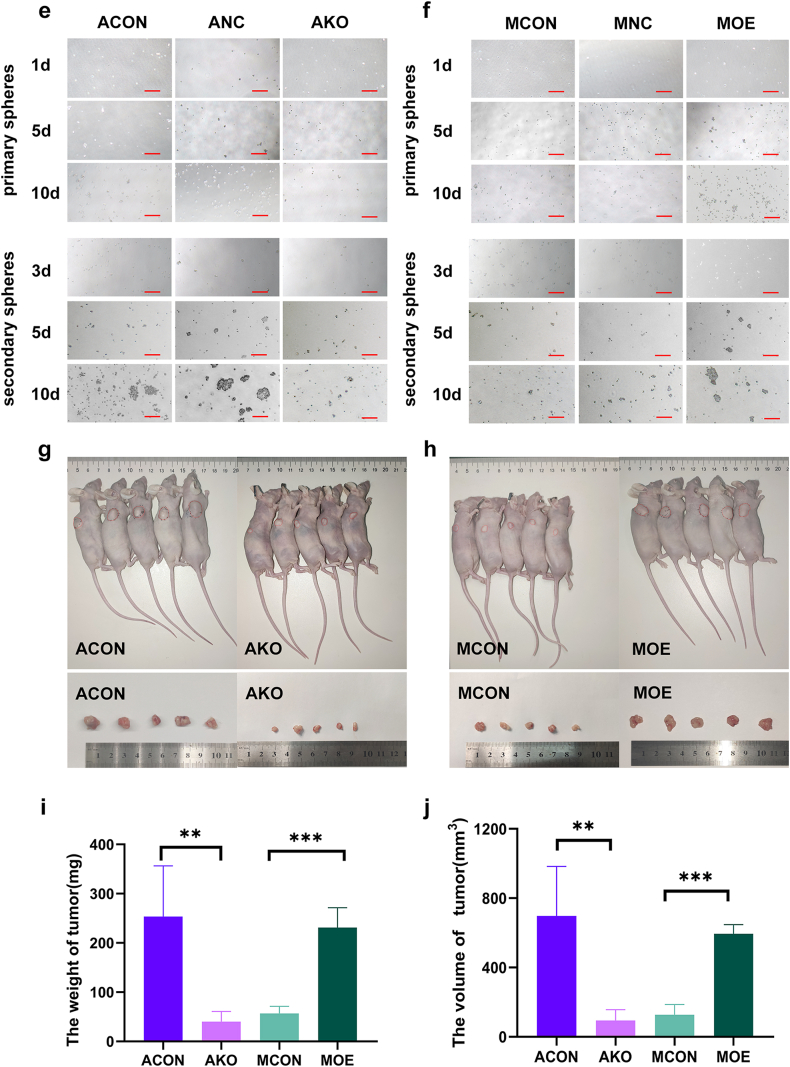
MSLN promoted the cancer stem cell traits. MSLN increased self-renewal ability as detected by plate-cloning(a,b), soft agar-cloning(c,d) and tumor sphere-forming ability(e-f). Scale bar = 800μm. Effect of MSLN on xenograft tumor growth 30 days after inoculation(g-j). The information is shown as mean ± SD from 5 repeated trials (*p < 0.05, **p < 0.01, ***p < 0.001, ****p < 0.0001).

**Table 6:** The weight and volume of xenograft tumor.

Group	Weight (g)	Volume (mm^3^)	Average (g)	*P* value	Group	Weight (g)	Volume (mm^3^)	Average (g)	*P* value
ACON-1	0.390	1158.07	0.254	*p* = 0.0019	MCON-1	0.065	181.11	0.057	*p* < 0.0001
ACON-2	0.311	793.86			MCON-2	0.035	69.57		
ACON-3	0.166	515.03			MCON-3	0.072	200.45		
ACON-4	0.260	537.53			MCON-4	0.052	96.04		
ACON-5	0.141	483.46			MCON-5	0.060	88.24		
AKO-1	0.024	54.82	0.040		MOE-1	0.192	576.35	0.231	
AKO-2	0.033	58.51			MOE-2	0.193	577.33		
AKO-3	0.074	198.49			MOE-3	0.225	524.67		
AKO-4	0.045	104.93			MOE-4	0.279	661.80		
AKO-5	0.024	55.79			MOE-5	0.266	632.05		

### 3.5 Pancreatic cancer cells with overexpression of MSLN exhibited resistance to gemcitabine

The cell viability curves of groups treated with different concentrations of gemcitabine for 48 h were obtained by CCK8 assay. IC50 of AKO was significantly smaller than that of ACON and ANC (Fig. 7a–d) significantly, while IC50 of MOE was significantly larger than that of MCON and MNC (Fig. 7e–h) significantly. The results of gemcitabine resistance of pancreatic cancer cells with overexpressing MSLN were shown in Table 7.

**Fig. 7:**
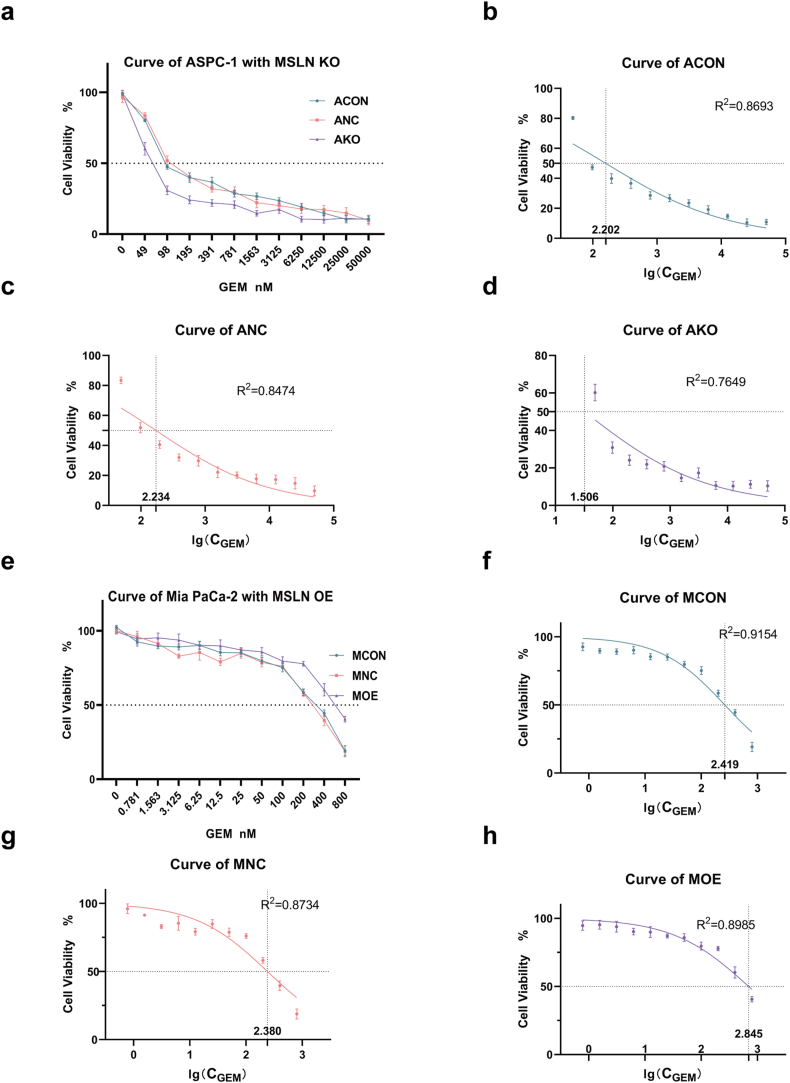
MSLN enhanced resistance of pancreatic cancer cells to gemcitabine. IC_50_ of AKO was smaller than that of ACON and ANC (a); the graph (b–d**)** represented nonlinear fit of cell viability and gemcitabine concentration for each cell line respectively. IC_50_ of MOE was larger than that of MCON and MNC (e); the graph (f–h**)** significantly represented nonlinear fit of cell viability and gemcitabine concentration for each cell line respectively. The data represented as mean ± SD of 6 replicated experiments.

**Table 7:** Estimation of IC_50_ by nonliner regression.

Group	Lg (IC_50_)	IC_50_ (nM)	95%CI (nM)	R squared
ACON	−0.7981	159.2	124.0–199.0	0.8693
ANC	−0.7657	171.5	132.7–215.6	0.8474
AKO	−1.494	32.0	18.6–47.8	0.7649
MCON	2.419	262.2	228.6–303.3	0.9154
MNC	2.380	240.0	200.2–292.0	0.8734
MOE	2.845	700.1	584.0–872.3	0.8985

## 4 Discussion

Pancreatic cancer has very poor prognosis, making its treatment very challenging [1]. At present, pancreatic cancer has been recognized as a systemic disease. Besides surgery, chemotherapy is crucial in the comprehensive treatment of pancreatic cancer [19]. Targeted therapy and immunotherapy have been explored extensively [20]. However, the overall efficacy of therapy is not satisfactory. Chemoresistance of pancreatic cancer confers poor clinical outcome. The resistance of pancreatic cancer to chemotherapy is influenced by both internal factors within the cancer cells themselves and external factors present in the pancreatic cancer mesenchyme. Intrinsically, the development of chemotherapy resistance in pancreatic cancer cells can be attributed to the activation or deactivation of transport mechanisms that neutralize gemcitabine, the generation of metabolites that competitively interfere with drug action, the induction of EMT, and stemness of cancer cells, among other factors. On the other hand, the extrinsic aspect of chemotherapy resistance typically revolves around the tumor stroma. The stroma of pancreatic cancer comprises various components such as extracellular matrix (ECM), cancer-associated fibroblasts (CAFs), pancreatic stellate cells (PSCs), inflammatory cells, immune cells (including tumor-associated macrophages), endothelial cells, nerve cells, and other cell types [21,22], all of which play a pivotal role in the development of chemotherapy resistance in pancreatic cancer [[23], [24], [25], [26]]. The dense stroma exerts pressure on blood vessels, creating a hypoxic environment that hampers the effective delivery of chemotherapy agents, thereby facilitating tumor progression [27]. In such circumstances, TME may induce immune suppression as a strategy to evade immune surveillance. Moreover, the stromal components and hypoxic conditions can stimulate EMT and contribute to gemcitabine resistance [28,29]. Chemoresistance is closely related to EMT [20], but molecular mechanism is not clear.

According to our previous study, pancreatic cancer highly expressed MSLN [11]. MSLN expression levels varied significantly among different pancreatic cancer cell lines [30]. It is unclear if MSLN is related to chemotherapy resistance of pancreatic cancer. Here, we comprehensively and systematically explored potential roles of MSLN in chemoresistance and its relationship with proliferation, EMT and cancer stem cell traits of pancreatic cancer cells. The expression level of MSLN varied greatly in different pancreatic cancer cell lines. Based on MSLN KO and OE models, we performed a variety of experiments to investigate potential effects of MSLN on intercellular-matrix adhesion, proliferation, migration, invasion and chemoresistance of pancreatic cancer cells.

Adhesion is mediated by adhesion molecules on the cell surface and can be divided into two categories: intercellular adhesion and intercellular-matrix adhesion. The later refers to tumor cells adhering to basement membrane and extracellular matrix components through membrane surface receptors, which can promote tumor immune escape and metastasis, as the initial step of invasion [31]. Combination of MSLN and MUC16 mediated adhesion in ovarian cancer [32]. Tumor progression involves the loss of E-cadherin function and transition to a more invasive phenotype, necessitates the controlled management of connections between cells and between cells and the surrounding matrix [33]. Interestingly, in our pre-experiment, there were few cells adhering to the bottom wall of the culture dish after 30 min or 1 h of incubation. We extended the incubation time and found that intercellular-matrix adhesion increased with time, and this increase was more pronounced in cells with high expression of MSLN. We concluded that MSLN could promote intercellular-matrix adhesion in pancreatic cancer.

Higher levels of MSLN expression in pancreatic cancer cells are associated with increased cell proliferation, similar to what has been observed in ovarian cancer [34], lung cancer [35] and malignant mesothelioma [15]. To date, there is evidence that cell cycle dysregulation can lead to uncontrolled proliferation and promote tumor development by initiating unplanned cell division [36]. By detecting proliferation and motility of tumor cells, invasion and metastasis can be indirectly determined [37]. Enhancement of cell proliferation directly led to tumorigenicity [38]. MSLN had a promotive effect on tumor growth of pancreatic cancer.

Migration was significantly inhibited when MSLN was knocked out, whereas enhanced when MSLN was overexpressed. MSLN was positively correlated with migration and invasion of pancreatic cancer cells, as described in ovarian cancer cells [31]. Metastasis contributes to poor prognosis in patients with pancreatic cancer. It requires multiple steps, including separating from the primary site, penetrating the physiological barrier, obtaining mobility, reaching and adhering to a distant site, colonizing and growing, and finally forming outgrowth [39]. MSLN could promote intercellular-matrix adhesion, proliferation, migration and invasion of pancreatic cancer cells, which might lead to distant metastasis. Early metastasis is attributed to loss of apical basal polarity and dissolution of adherence junction proteins, disruption of tight junctions, and acquisition of mesenchymal cell characteristics, namely EMT [31,40]. EMT was characterized by enhanced migration and invasion [41,42], so we speculated that MSLN could promote EMT process in pancreatic cancer.

Currently, EMT was considered as an important step of cancer progression [43]. Cells lost polarized epithelial phenotype and acquired mesenchymal traits [44]. E-Cadherin was a characteristic protein in epithelial cells. EMT was accompanied by loss of E-Cadherin and up-regulation of mesenchymal markers including Vimentin and Snail [[45], [46], [47]]. The phenotypes of mesenchymal and epithelial cells could be transformed into each other under specific microenvironment [48]. EMT help cancer cells to evade immune system and to promote metastasis [49,50]. The relationship between EMT and tumor cell invasion and metastasis is one of the focuses of tumor research. Our results showed that up-regulation of MSLN was accompanied with the expression changes of E-Cadherin, Vimentin and Snail in pancreatic cancer cells, hence promoting EMT. MSLN KO exhibited opposite functions. MSLN plays a key role in promoting migration and invasion of tumor cells through EMT. This verifies our hypothesis that MSLN can promote EMT, thus metastasis of pancreatic cancer.

EMT has the ability to trigger tumor cells to acquire characteristics similar to pluripotent stem cells, including self-renewal, proliferation, and migration, ultimately supporting the continuous growth of the tumor. Cancer cells displaying characteristics of stem cells increased the formation of colonies and tumorigenic potential [51]. Cell cloning usually referred to ancestral cell division and reproduction to form cell lines with the same genetic characteristics. In two-dimensional and three-dimensional cell cultures and animal models with MSLN KO and MSLN OE, our results showed MSLN could promote tumorigenicity. Notably, MSLN knockout inhibited proliferation, sphere formation and anchor-independent growth, as well as the intercellular-matrix adhesion, migration and invasion of cancer cells, which suggested that MSLN promoted the EMT process of pancreatic cancer cells, and participated in the regulation of the cancer stem cell-like properties. The stemness of tumor cells can promote chemoresistance in terms of cell cycle [52,53], DNA damage repair [54], drug efflux mechanism [55], and EMT [56,57]. Therefore, we believe that MSLN promotes chemoresistance of pancreatic cancer by regulating EMT and tumor cell stemness. To explore the mechanism of MSLN promoting EMT and its relationship with chemotherapy resistance, and screen effective targets may be a breakthrough to address the poor prognosis of pancreatic cancer.

Resistance to chemotherapy in pancreatic cancer is a significant, unresolved medical issue [58]. Similarly, mesothelioma and colorectal cancer patients with high expression of MSLN are prone to chemotherapy resistance [59,60]. Amatuximab, an MSLN antibody, increased the sensitivity of pancreatic cancer cells to gemcitabine [61]. We have found that MSLN can regulate EMT of tumor cells, associated with chemotherapy resistance and pancreatic cancer cells with high levels of MSLN have stronger proliferation and invasion to promote tumor development. Thus, targeting MSLN may be an effective strategy to inhibit tumor progression. Indeed, MSLN KO proved to be highly efficient. However, we have not yet verified these observations in vivo. We will explore other biological functions of MSLN in pancreatic cancer in future studies.

## 5 Conclusion

In conclusion, MSLN could induce chemoresistance by enhancing migration, invasion, EMT and cancer stem cell traits of pancreatic cancer cells. Targeting MSLN could represent a promising therapeutic strategy for reversing EMT and chemoresistance in pancreatic cancer cells.

## Ethics statement

The entire experimental process followed specific guidelines for Animal Experiments at Beijing Shijitan Hospital, Capital Medical University with the approval of Institutional Animal Care and Use Committee (sjtkyll-lx-2022(069)).

## Data availability statement

The researchers verify that the information backing the conclusions of this research can be found in the article and its additional resources.

## Funding

This work was supported by the scientific research fund project of Beijing Municipal Education Commission [grant number: KM201710025025].

## CRediT authorship contribution statement

**Jili Hu:** Writing – review & editing, Writing – original draft, Software, Methodology, Formal analysis, Data curation. **Jia Wang:** Writing – review & editing, Writing – original draft, Methodology, Data curation, Conceptualization. **Xu Guo:** Software, Methodology. **Qing Fan:** Data curation, Conceptualization. **Xinming Li:** Software, Methodology. **Kai Li:** Software, Methodology. **Zhuoyin Wang:** Visualization, Validation, Software. **Shuntao Liang:** Writing – review & editing, Software, Methodology. **Buhe Amin:** Supervision, Methodology, Formal analysis. **Nengwei Zhang:** Validation, Conceptualization. **Chaowen Chen:** Project administration, Conceptualization. **Bin Zhu:** Writing – review & editing, Supervision, Resources.

## Declaration of competing interest

The authors declare that they have no known competing financial interests or personal relationships that could have appeared to influence the work reported in this paper.

## Figures and Tables

**figs1:**
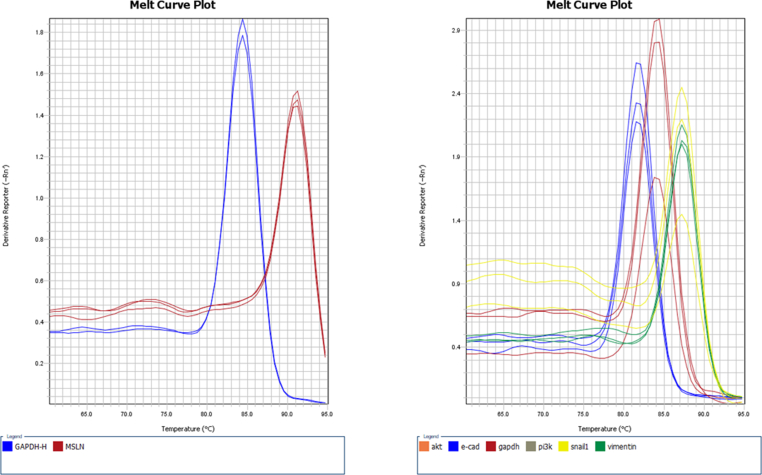


**figs2:**
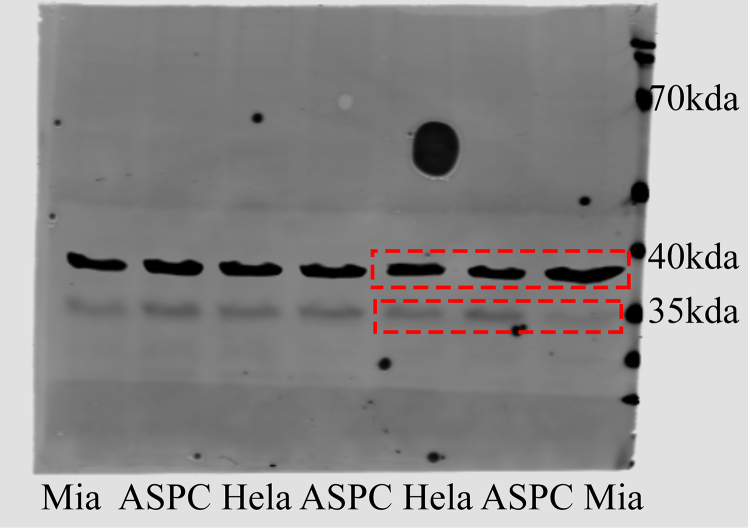


**figs3:**
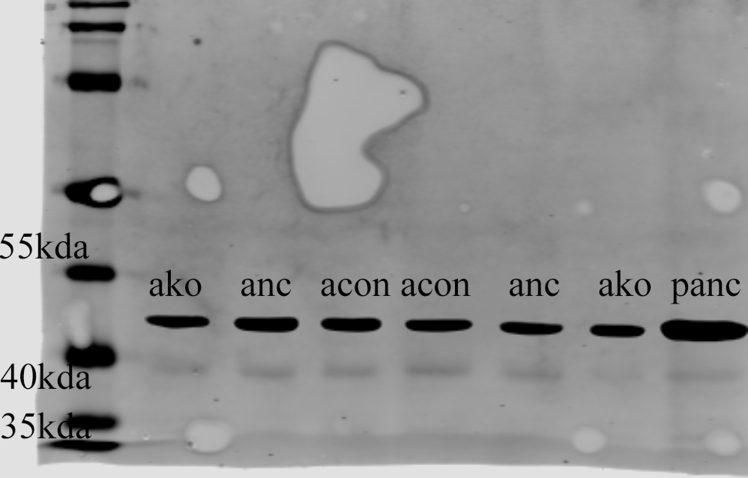


**figs4:**
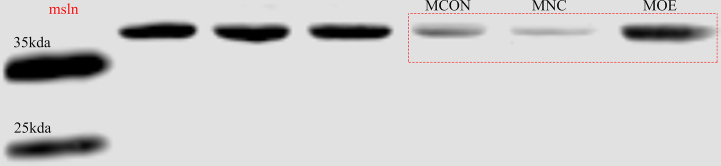


**figs5:**
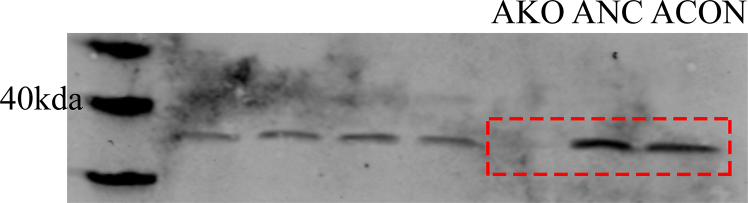


**figs6:**
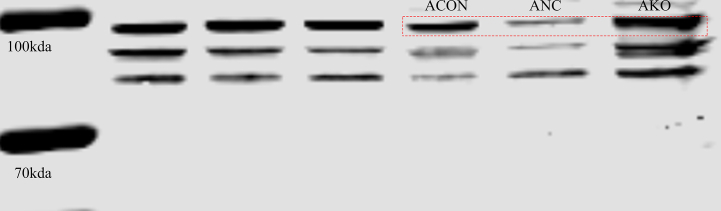


**figs7:**
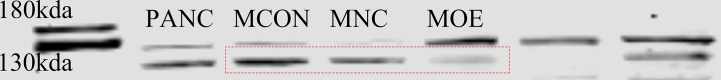


**figs8:**
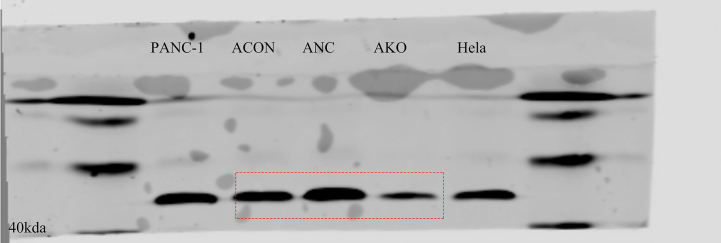


**figs9:**
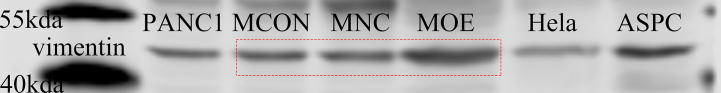


**figs10:**
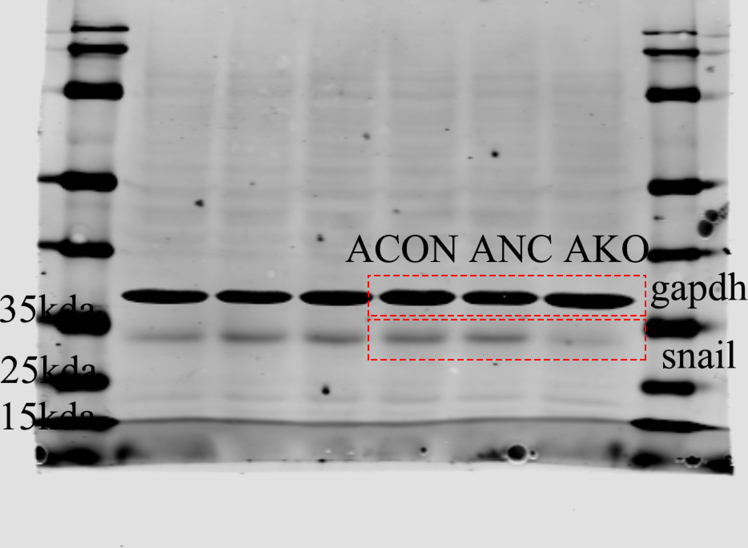


**figs11:**